# Inhibition of NF-kB 1 (NF-kBp50) by RNA interference in chicken macrophage HD11 cell line challenged with *Salmonellaenteritidis*

**DOI:** 10.1590/S1415-47572009000300013

**Published:** 2009-09-01

**Authors:** Hsin-I Chiang, Luc R. Berghman, Huaijun Zhou

**Affiliations:** Department of Poultry Science, Texas AM University, College Station, TXUSA

**Keywords:** RNA interference, NF-kBp50, macrophage, *Salmonella*, chicken

## Abstract

The NF-kB pathway plays an important role in regulating the immunity response in animals. In this study, small interfering RNAs (siRNA) were used to specifically inhibit NF-kB 1 expression and to elucidate the role of NF-kB in the signal transduction pathway of the *Salmonella* challenge in the chicken HD11 cell line. The cells were transfected with either NF-kB 1 siRNA, glyceraldehyde 3-phosphate dehydrogenase siRNA (positive control) or the negative control siRNA for 24 h, followed by *Salmonella enteritidis* (SE) challenge or non-challenge for 1 h and 4 h. Eight candidate genes related to the signal pathway of SE challenge were selected to examine the effect of NF-kB 1 inhibition on their expressions by mRNA quantification. The results showed that, with a 36% inhibition of NF-kB 1 expression, gene expression of both Toll-like receptor (TLR) 4 and interleukin (IL)-6 was consistently and significantly increased at both 1 h and 4 h following SE challenge, whereas the gene expression of MyD88 and IL-1β was increased at 1 h and 4 h, respectively. These findings suggest a likely inhibitory regulation by NF-kB 1, and could lay the foundation for studying the gene network of the innate immune response of SE infection in chickens.

## Introduction

*Salmonella enterica* serovar Enteritidis (SE) has become one of the most common *Salmonella* serotypes in many countries (Braden, 2006). Epidemic SE emerges as /constitutes the main source of salmonellosis in humans through the consumption of contaminated poultry or shell eggs. SE can persist in the cecum or ovaries of adult birds for months without triggering clinical signs. Colonizing SE can continue to be excreted in faces (horizontal transmission) or through the yolk (vertical transmission) to contaminate other birds in the flock, as well as poultry products such as meat (after slaughtering) and eggs (Tilquin *et al.*, 2005).

Current control of salmonellosis in poultry is mainly through hygiene measures combined with vaccination programs, although the present vaccines are only partially effective (Wigley *et al.*, 2002). Selection of chickens for genetic resistance to *Salmonella* infection offers an alternative environment-friendly control measure. Macrophages are critical components of the immune system and play significant roles in both innate and acquired immune responses during SE infection. Through the process of phagocytosis, the macrophage is responsible for the clearance and destruction of both intracellular and extracellular pathogens (Lavric *et al.*, 2008; Ohl and Miller, 2001; Zhang *et al.*, 2008). Previous studies on chickens have shown that macrophages from a *Salmonella*-resistant line have greater capability for clearing colonizing *Salmonella* than macrophages from susceptible lines (Wigley *et al.*, 2002). The different biology of macrophages might be associated with greater and more rapid expression of pro-inflammatory cytokines generated through the NF-kB signal pathway (Wigley *et al.*, 2006).

Although gene expression profiling of chicken macrophages has already been undertaken using an avian macrophage-specific cDNA micro-array with lipopolysaccharide (LPS) stimulation (Bliss *et al.*, 2005) , the role of NF-kB in the immune response to SE infection in chickens is still unknown. NF-kB is composed of members of the v-rel reticuloendotheliosis viral oncogene homolog (Rel) family. A total of five NF-kB proteins have already been identified in chickens, namely NF-kB 1 (p50/p105), NF-kB 2 (p100/p52), RelA (p65), RelB and c-Rel. Given that NF-kB is the central regulator of the innate immune response to invasive bacteria, and that it is activated by MyD88-dependent signaling of the TLR pathway (Elewaut *et al.*, 1999; Moynagh, 2005), it is essential to elucidate the role of NF-kB in the TLR pathway in macrophages and to further our knowledge on SE pathogenesis in chickens. To do so, RNA interference (RNAi) technology using chemically synthesized siRNA was applied to specifically inhibit NF-kB expression in a chicken macrophage cell line (HD11). Expressions of selected genes including receptors, adaptors and cytokines associated with NF-kB pathway were evaluated, both before and after SE challenge in the siNF-kB 1-treated cells. In the present study, gene expression of several candidate genes was influenced by the NF-kB 1 inhibition.

## Material and Methods

###  Culture of chicken macrophage HD11 cells

Chicken macrophage HD11 cells, constituting an established chicken myelomonocytic line transformed by the *myc-*encoding MC29 virus (Van 1996), were used in this study. The HD11 cells were grown in Dulbecco's Modified Eagle's Medium (Sigma-Aldrich, St. Louis, MO), supplemented with 1% sodium pyruvate (Sigma-Aldrich, St. Louis, MO), 1% penicillin/streptomycin (Sigma-Aldrich, St. Louis, MO), 1% glutamax (Invitrogen, Carlsbad, CA), 5% chicken serum (Sigma-Aldrich, St. Louis, MO) and 5% fetal-bovine serum (Atlanta biologicals, Lawrenceville, GA) at 37 °C in a 5% CO_2_ incubator, following routine cell-culture procedures.

###  siRNA synthesis and transfection

Messenger RNA sequences of chicken NF-kB 1 (NM_205134) and glyceraldehyde 3-phosphate dehydrogenase (GAPDH, NM_204305.1) were used to design siRNA primers as target genes and positive controls, respectively. For each mRNA sequence, three different siRNA primers were designed for targeting distinct sites in order to compare silencing efficacy. All of the siRNA primers were synthesized and annealed by Ambion (Ambion, Austin, TX) (Table 1). A universal negative control siRNA primer (Ambion, Austin, TX), with no specific targeting to the chicken genome, was used to normalize relative gene inhibition of the target gene. Twenty-four hours before transfection, 450 μL HD11 cells were transferred onto a 24-well plate (pre-plating) to reach 50 to 80% confluence, to then be transfected with either NF-kB 1 siRNA (siNF-kB 1) or GAPDH siRNA (siGAPDH) or a scrambled siRNA negative control (siNC), using the chemical transfection reagent siPORT-Amine (Ambion, Austin, TX). Transfection was undertaken according to the manufacturer's manual, with minor modifications. Briefly, 1.5 μL of 10 μM annealed siRNA were thoroughly mixed and incubated with 4 μL of siPORT-Amine in a volume of 50 μL of Opti-mem medium (Invitrogen, Carlsbad, CA) at room temperature for 20 min, this then being added to 450 μL of HD11 cell culture with gentle agitation for mixing. The conditions for siRNA transfection were optimized by adjusting different transfection parameters, including cell number (2x10^5^, 3x10^5^ and 4x10^5^ cells/mL), siPORT-Amine concentration (3, 4, and 5 uL/50 uL of total reaction volume of siRNA mixture), siRNA concentration (20 nM, 30 nM, and 40 nM) and incubation time (12 h, 24 h, 36 h, and 48 h). After incubation, the total RNA of siRNA- (siNF-kB 1, siGAPDH or siNC) treated cells was extracted for first strand cDNA synthesis, relative gene inhibition being measured by real-time quantitative PCR (qRT-PCR) using SYBR green master mix and the ABI prism 7900HT system (Applied Biosystems, Foster, CA).

###  SE challenge

An SE isolate (no. 97-11771, kindly provided by Dr. Kogut at College Station, USDA-ARS) was used in the present study. The SE was maintained in glycerol stocks at -80 °C, and grown overnight in Luria-Bertani broth at 37 °C to recover the fresh culture. The recovered SE was adjusted in sterile PBS to a concentration of 1x10^9^ CFU per milliliter, using spectrophotometric absorbance as previously described (Ferro *et al.*, 2004). Before the challenge, the HD11 culture medium was replaced by an antibiotic-free medium and the cells cultured for 2 h prior to challenge. The number of siRNA-treated HD11 cells was calculated from synchronized duplicates of the siNC transfected group. Both siNF-kB 1 and siNC transfected HD11 cells were stimulated with non-opsonized SE at a multiplicity of infection (MOI) of 100, or with sterilized PBS (non-challenged). The treated culture plate was then centrifuged at 1000 x g for 5 min to maximize the contact between bacteria and cells, and then incubated at 37 °C, 5% CO_2_ for 1 h or 4 h as previously described (Wigley *et al.*, 2002).

In this study we did not carry out phagocytosis assays to compare mock-transfected and NF-kB transfected cells. However, a previous similar study has demonstrated that there was no appreciable influence on the phagocytic capability of CpG-treated HD11 cells that were SE stimulated at different time points (Xie *et al.*, 2003).

###  Quantitative RT-PCR

Total RNA was extracted from the SE-challenged and non-challenged cells with a RNAqueous kit (Ambion, Austin, TX), according to manufacturer's instructions. The cDNA was synthesized from equal amounts (300 ng) of total RNA with a random hexamer primer from a Thermoscript RT-PCR system kit (Invitrogen, Carlsbad, CA) according to instructions. The cDNAs from different treatments were quantified by qRT-PCR using the ABI prism 7900HT system (Applied Biosystems, Foster, CA) with software setting at the relative quantification mode. Briefly, the 20 μL reaction mixtures contained 10 μL of SYBR Green PCR Master Mix (Applied Biosystems, Foster, CA), 0.3 μM of each specific oligonucleotide primer (Table 2) and 1 μL of non-diluted first strand cDNA synthesized from 300 ng of total RNA. The conditions for qRT-PCR amplification were set up as: 1 cycle at 95 °C for 10 min, 40 cycles at 95 °C for 15 s and 59 °C for 1 min. Dissociation curves were obtained for each amplified product at the end of amplification. Each individual sample was run in triplicate and the average critical threshold cycle (Ct) used for data analysis. The Ct values of target genes were normalized by the Ct value of internal control (chicken β-actin gene). Within the group of a same siRNA treatment (siNF-kB 1 or siNC), the normalized Ct value (ΔCt) from SE-challenged HD11 cells was compared to the ΔCt from non-challenged cells, the difference (ΔΔCt) being transformed into 2^-^^Ct^ value as the estimated fold change of the siNF-kB 1 effect. The relative gene expressions (SE-challenged to non-challenged) were represented by fold change at both 1 h and 4 h post-infection, and the comparisons of fold change between siNC and siNF-kB 1 treated HD11 cells were carried out at different time points.

###  Statistical analysis

Data analysis was undertaken by means of ΔΔCt from six individual data points in two biological replicates, the mutual difference being evaluated by two tailed, paired Student's *t*-tests using the Microsoft^®^ Excel 2003 version (Microsoft Corporation, 2003), p < 0.05 being considered significant. Data displayed in the Figures were expressed as the means of fold change ± standard error from six individual data points.

## Results

###  RNAi efficacy of siGAPDH and siNF-kB 1 in HD11 cells

**Figure 1:**
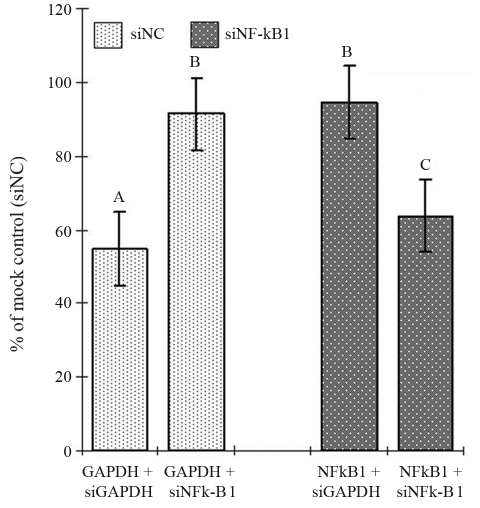
Reduced mRNA expression (GAPDH and NF-kB 1) in chicken HD11 cells after siGAPDH or siNF-kB 1 treatments. The data are presented as the mean (± standard error) from two replicate experiments. Data with different superscripts are statistically different (p < 0.05).

Since transfection efficacy of siRNA delivery varies in different target cell types, and in order to establish a protocol for siRNA in the HD11 cell line, a positive control siRNA (chicken siGAPDH) was first used to optimize transfection conditions. Three distinct siGAPDH primers (data not shown) were transfected with different primer concentrations, transfection reagent concentrations and HD11 cell concentrations, with varying periods for incubation. The optimal transfection condition was defined as the condition under which siGAPDH could induce the highest inhibition of chicken GAPDH expression. In the present study, the optimized condition were as follows: cell concentration of 2.4 x 10^5^/mL, siPORT-Amine (non-diluted): siRNA (10 μM) at 2.6:1 (v/v), with an incubation period of 24 h, in which GAPDH expression was reduced by 45%, when compared to the mock transfected group using negative control siRNA primers (Figure 1). The same transfection conditions were then used for delivering siNF-kB 1 primers, similar efficacy of inhibition (36%) being observed in the NF-kB1 gene. A cross test was set up for measuring GAPDH expression with a siNF-kB 1 treated sample and NF-kB 1 expression with a siGAPDH treated one. Only small reductions of gene expression (8% for GAPDH and 5% for NF-kB 1) were observed within the cells treated with target-unrelated siRNAs. The siRNA primers with the highest inhibitory efficacy (one out of three from siGAPDH primers and one out of six from siNF-kB 1, data not shown) were selected for silencing target genes in SE-challenged HD11 cells (Table 1).

###  The effects of reduced NF-kB 1 expression on SE induced immune response

**Figure 2:**
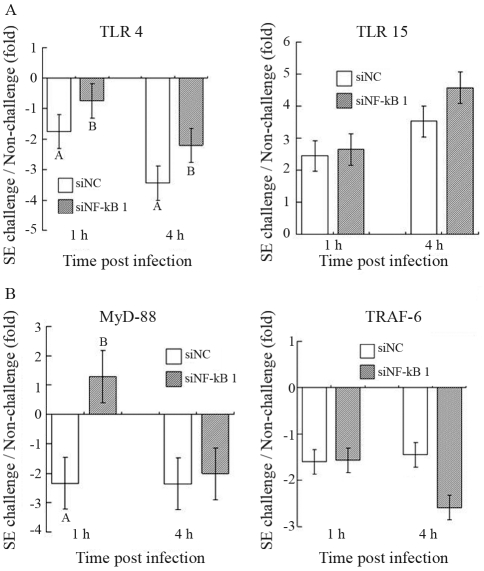
The effects of NF-kB 1 inhibition on mRNA expression of (A) receptors (TLR4, TLR15) and (B) adaptors (MyD88, TRAF6) from HD11 cells at different points in time, post SE infection. The genes with positive fold changes are up-regulated. The genes with negative fold changes are down-regulated. The data are presented as the mean (± standard error) from two replicate experiments. Data with different superscripts at the same time point are statistically different (p < 0.05).

Both siNF-kB 1 treated and negative control (siNC treated) HD 11 cells were followed by SE challenge. The expressions of several candidate genes, including receptors, adaptors, and cytokines, were measured before and after SE challenge (1 and 4 h post-infection). Both TLR4 and TLR15 were receptor candidate genes due to their critical functions of microbial recognition by binding pathogen-associated molecular patterns (PAMPs). With SE challenge, TLR4 expression was down-regulated at both 1 h (-1.75 - fold) and 4 h (-3.45 - fold) post-infection (Figure 2A). Interestingly, the inhibition of siNF-kB 1 was found to significantly increase down-regulated TLR4 gene expression at both time points (p < 0.05). Unlike TLR4, the expression of TLR15 was consistently up-regulated with SE challenge (2.44 - fold at 1 h and 3.52 - fold at 4 h post-infection), whereas no significant change was observed in siNF-kB 1 inhibition. Myeloid differentiation primary response gene 88 (MyD88) and TNF receptor-associated factor 6 (TRAF6) genes are important adaptors that assist signal transduction in the canonical TLR pathway. Within siNC treated groups, both MyD88 and TRAF6 were consistently down-regulated with SE challenge (-2.34 - fold at 1 h and -2.37 - fold at 4 h, post-infection for MyD88, and -1.60 - fold at 1 h and -1.45 - fold at 4 h post-infection for TRAF6) (Figure 2B). Nevertheless, with NF-kB 1 inhibition, MyD88 was significantly (p = 0.0047) up-regulated 1.28 - fold at 1 h post-infection, whereas no significant change was found in TRAF6 expression.

**Figure 3:**
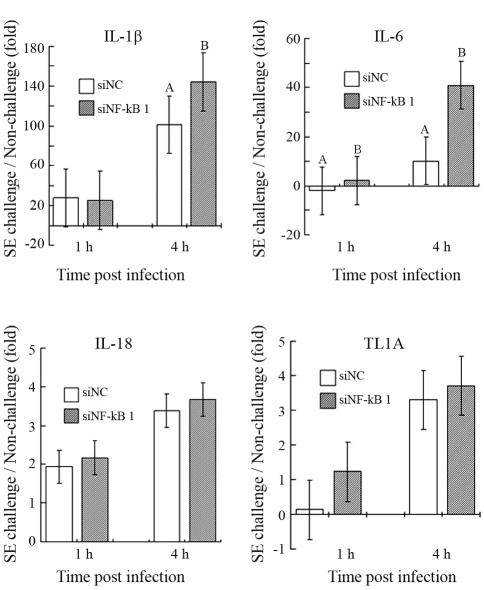
The effects of NF-kB1 inhibition on mRNA expression of cytokines (IL-1β, IL-6, IL-18 and TL1A) in HD11 cells at different time point post SE infection. The genes with positive fold changes are up-regulated. The genes with negative fold changes are down-regulated. The data are presented as the mean (± standard error) from two replicate experiments. Data with different superscripts at the same time point are statistically different (p < 0.05).

Four cytokine genes were chosen to examine the effects of inhibited NF-kB 1 on SE induced immune response. All other cytokine expressions were up regulated after SE challenge except that down regulated expression was observed at 1 h post-infection in IL-6. Furthermore, gene expressions were consistently higher at 4 h than 1 h post-infection (Figure 3). The comparison between siNC and siNF-kB 1 treated samples indicated that IL-1β expression was significantly (p = 0.001) up-regulated by siNF-kB 1 inhibition only at 4 h post-infection, whereas IL-6 expression was significantly up-regulated at both 1 h and 4 h post-infection (p = 0.009 and p = 0.0004, respectively). No significant effect of siNF-kB 1 inhibition on IL-18 or TL1A was observed.

## Discussion

RNAi is the process of sequence-specific post-transcriptional gene silencing. The mediators of messenger RNA degradation are 21~26 nucleotide siRNAs generated by a ribonuclease III-like dicer cleaving the longer double strand RNA (dsRNA) (Duxbury and Whang, 2004; Finnegan and Matzke, 2003). RNAi offers great potential for both *in vitro* target validation and novel therapeutic strategies, based on its mechanism that permits selective inhibition of specific gene expression (Aigner 2006). One of the key experimental approaches to elucidate gene function is to selectively ablate its expression or activity by the loss-of-function (LOF) approach. The gene-knockout approach is not currently applicable in chickens. RNAi using siRNA is more favorable in terms of efficiency, efficacy, and cost compared to other LOF techniques such as antisense DNA oligonucleotides, small molecular inhibitors, and dominant-negative mutants technology (Aigner 2006; Dees *et al.*, 2000; Roth 1986). Along with these features, siRNA has less chance of triggering the interferon response than long double-stranded RNA (Stark *et al.*, 1998). Therefore, siRNA became the first option for LOF selected in the present study.

Most currently reported RNAi experiments in chickens were focused on studying embryogenesis using vector-based RNAi transfection (Chesnutt and Niswander 2004; Das *et al.*, 2006; Kudo and Sutou 2005), and very few studies reported using chemically synthesized short interfering RNAs (siRNAs) on chicken cells (Hu *et al.*, 2002; Sato *et al.*, 2006). The lack of information regarding validated positive and negative control siRNA in chickens makes it more difficult to conduct an effective siRNA experiment using the chicken as a model. Since siRNA gene silence is a transfection-dependent technology, optimization of siRNA transfection was considered the first step to set up a successful siRNA experiment (Duxbury and Whang 2004). We found that the 24-well plate was the most suitable for condition optimization and was capable of providing enough RNA extracted from HD11 cells for mRNA quantification when using qRT-PCR. It has been reported that the efficiency of siRNA transfection is related to intrinsic thermodynamic properties and target site accessibility of the siRNA duplex (Kurreck 2006). In spite of many successful RNAi mediated approaches having been reported, the design of highly potent siRNAs still remains an obstacle. Currently, the approach using a multiple (three or more) primer design targeted to different sites in the target sequence is widely used to overcome this hurdle (Duxbury and Whang, 2004). By using Ambion's design algorithms, our results showed that about 30% of tested siRNA primers could achieve a reproducible gene knockdown efficacy of around 40% compared to the siNC groups, which implies that designing three pairs of siRNA primers for one mRNA sequence might be an efficient and economic approach. Although siRNAs are not thought to trigger general translational attenuation through interferon response, as long double-stranded RNA does, the specificity of silencing ability and potential side effects (*e.g.* transfection reagent toxicity) were tested by measuring expression changes in target-unrelated genes. The small reduction in target-unrelated genes in the present study (data not shown) implied that there was no appreciable general translational attenuation, thereby indicating that the observed effects were not biased. In contrast, both target gene and positive control showed a significant reduction in gene expression with the corresponding siRNA treatments. Since the expression of all candidate genes in the SE-challenged cells was measured within 6 h after NF-kB 1 inhibition, recovery of repressed NF-kB 1 in the present study was unlikely, due to the very short time span allowed for HD 11 cell division.

The macrophage is one of the principle leukocytes involved in defending epithelium cells against *Salmonella* infection (Ohl and Miller 2001). Macrophages recognize various microbial patterns by many receptors including Fc and complement receptors, integrins, lectins, the mannose receptor, CD14, and the TLRs (Underhill and Ozinsky 2002). One of the most extensively described pathways is the TLR. The predominant signaling pathway used by TLRs results in the activation of NF-kB, a transcription factor that is involved in both T-cell activation and the production of cytokines that promote the extermination of invading microbes (Moynagh, 2005). According to the mammalian model, NF-kB was assumed to participate in signal transduction of the TLR (MyD88- dependent) pathway besides cytokine release in response to bacterial infection (Lynn *et al.*, 2003). However, no direct connection has been confirmed between NF-kB and associated genes involved in the TLR pathway in chickens. In the present study, the role of NF-kB in the TLR pathway was studied by combining NF-kB 1 silencing and SE stimulation of HD11 cells. Eight candidate genes involved in the TLR pathway were analyzed after NF-kB 1 repression. It is assumed that genes with a significant change in expression might have a stronger connection with the NF-kB signal pathway.

Although in chickens LPS can be sensed via the TLR4 receptor, the role of chicken TLR4 in *Salmonella* infection has been contradictory (Keestra and van Putten 2008; Wigley 2004). Allelic variation in TLR4 was associated with susceptibility to *S*. ser. Typhimurium in chickens (Leveque *et al.*, 2003), whereas Higgs and his colleagues reported that there was no up-regulation of TLR4 in the cecum following *S.* Typhimurium infection (Higgs *et al.*, 2006). Very few studies have reported TLR4 gene expression in response to SE infection in chicken macrophages or HD11 cells. Our results showed down-regulation of TLR4 after SE challenge at both 1 h and 4 h post-infection, this being significantly attenuated following the inhibition of NF-kB 1 expression. For TLR15, a novel chicken TLR associated with *S.* Typhimurium infection (Higgs *et al.*, 2006), there was no statistical significance at both 1 h and 4 h post-infection, although a slight up-regulation with the inhibition of siNF-kB 1 was observed. The results implied that NF-kB1 might be involved in feedback mediating TLR4 expression through an inhibitory effect, but with no or only minor effects on mediating TLR15 expression.

Based on a MyD88 knock-out mice study (Janssens and Beyaert 2002; O'Neill 2003), it has been found that MyD88 is a universal adaptor for all TIR-domain-containing receptors except TLR-3. After TLRs are activated by their ligand, MyD88 recruits IL-1 receptor-associated kinases (IRAKs) to interact with the TLRs. These activated IRAKs then associate with TRAF6 to activate the IkB kinase (IKK) complex, which finally releases NF-kB by degrading the IkBα molecule (Takeda and Akira 2004; Yamamoto *et al.*, 2004). Both MyD88 and TRAF6 are downstream adaptors of TLR4, and interestingly, they both showed a consistent down-regulation with SE challenge. However, with the inhibition of siNF-kB 1, a dramatic up-regulation of MyD88 at 1 h post-infection was observed, while no significant change was found for TRAF6, except for an even lower expression at 4 h post-infection for some unknown reason. Few studies have reported the detailed regulation of MyD88 or TRAF6 in chickens. In human monotypic THP-1 cells, no significant change of mRNA expression on MyD88 was observed between 0 to 4 h after LPS stimulation (Tamai *et al.*, 2003). The stimulation of LPS on murine macrophages also showed no significant change in TRAF6 protein levels (Chen *et al.*, 2006). It has been reported that most adaptors function with subtle gene expression in transcriptome during signaling transduction (Ben-Shaul *et al.*, 2005). It is unclear whether the up-regulation of MyD88 in the present study is associated with variant chicken MyD88 isoforms or not (Qiu *et al.*, 2008). However, this is the first study to demonstrate adaptor gene expression change in chicken macrophages as a consequence of SE challenge. Our results also indicate a potential feedback regulation between MyD88 and NF-kB 1.

Inflammatory cytokines, such as IL-1β, IL-6, IL-12, IL-18, and TNF-alpha, are the end products of the MyD88-dependent TLR pathway (Takeda and Akira, 2004; Yamamoto *et al.*, 2004). The production of inflammatory cytokines would be expected in those macrophages where an inflammatory response occurs (Kaiser *et al.*, 2004). In the present study, all cytokines showed significantly up-regulated expression at 4 h post-infection, thereby indicating that SE challenge was sufficient to induce an innate immune response in HD11 cells. More rapid and dramatic expressional fold changes have already been observed for IL-1β (25 to 44 - fold) and IL-6 (-1.9 to 40 - fold), results similar to those reported from a previous study on chicken macrophages with *Salmonella* challenge (Wigley *et al.*, 2006). Although the synthesis of inflammatory proteins is assumed to be related to NF-kB binding to their cognate promoter, our results showed that repressed NF-kB has significant up-regulatory effects on both IL-1β and IL-6. This was not exactly what we expected. The possible reasons for these unexpected results could be: 1) The regulation of pro-inflammatory cytokines by a NF-kB pathway is modulated by the canonical NF-kB heterodimer (p50-p65). The knock-down of one of the components in this heterodimer might not be sufficient to reduce gene expression in these cytokines. This has prompted us to consider including the knock-down of another NF-kB component in our next study; 2) The induction of gene expression in these cytokines might be regulated by another transcription factor, such as AP-1, since AP-1 has been shown to induce cytokine expression along with that of NF-kB (Kogut *et al.*, 2008); 3) It is possible that the binding site of the heterodimer p50-p65 could also be occupied by the homodimer p50-p50, whereupon p50-p50 may function as a repressor to regulate p50-p65's role as a transcription factor essential for immune response (Beinke and Ley, 2004; Tong *et al.*, 2004). In mammals, IL-1β and IL-6 are both critical for activating the immune response and synthesizing acute-phase proteins (Giansanti *et al.*, 2006). It is speculated that these two pro-inflammatory cytokines might be essential in the early phase of the inflammatory stage against *Salmonella* infection.

The function of IL-18 is associated with enhancing a Th1-type response, besides activating PMN (polymorphonuclear) cells such as neutrophils in mammal (Lee *et al.*, 2004). Swaggerty and her colleagues reported that higher IL-18 mRNA expression in heterophils in chickens (a counterpart of neutrophils in birds) was associated with resistance to SE infection. This suggested that IL-18 may also play a protective role against *Salmonella* infections (Swaggerty *et al.*, 2006). A previous study in humans reported that repression of IL-18 has weak or absent repression with the competitive inhibition of NF-kB when compared to IL-1β (Lee *et al.*, 2004). In the present study, a rapid increase of IL-18 in chicken macrophages after SE challenge was observed. Notwithstanding, there is no significant effect on IL-18 gene expression with the inhibition of NF-kB 1.

Chicken TL1A (also known as TNF superfamily 15) was suggested to function as a substitute for mammalian TNF-α induced by LPS via the NF-kB pathway (Hong *et al.*, 2006; Takimoto *et al.*, 2005), although very few studies have addressed this novel chicken cytokine. Although no significant effect on TL1A expression was observed after NF-kB 1 repression, our results showed that SE challenge could also induce TL1A expression in chicken macrophages, especially 4 h post-infection. Increased TL1A may exert an inflammatory function by enhancing nitric oxide (NO) production and heterophil phagocytosis (Takimoto *et al.*, 2005).

Notably, all of the regulatory directions of siNF-kB 1 on cytokines were similar to its effects on TLR genes, supporting the possible function of NF-kB1 as an inhibitory component in the TLR pathway. In mammals, three pathways were reported as activating the NF-kB function through different dimeric complexes (p65-p50, p50-p50 and p52-RelB). The canonical pathway via p65-p50 heterodimers is essential for an immune response, whereas p50-p50 homodimers may function as repressors by competing for the DNA binding site of other NF-kB dimers (Lernbecher *et al.*, 1993). It was also reported that the adenovirus-mediated induced p50-p50 dimer displayed suppressed IL-1β activation, but had no effect on TNF-alpha in colon-derived HT-29 cells (Tong *et al.*, 2004). The novel findings in the present study have provided new insights into how NF-kB 1 can regulate the TLR signal transduction pathway as a possible inhibitory factor in chickens. Further studies focusing on RelA protein may help to reveal the roles of NF-kB in this sophisticated TLR signal pathway.

## Figures and Tables

**Table 1:** List of chemically synthesized small interfering RNAs for specific gene silencing.

Primer name	mRNA target	Sense / Anti-sense	Primer sequence	Accession n. in GenBank
siGAPDH	GAPDH	Sense	5'-GGUGCUGAGUAUGUUGUGGtt-3'	NM_204305
		Anti-sense	5'-CCACAACAUACUCAGCACCtg-3'	
siNF-kB 1	NF-kB 1	Sense	5'-GGAGAGGAUCCGUAUAUUAtt-3'	NM_205134
		Anti-sense	5'-UAAUAUACGGAUCCUCUCCtg-3'	

**Table 2:** List of primers for quantitative real-time RT-PCR analysis.

mRNA target		Primer sequence	Accession n. in GenBank	PCR product size (bp)
β -actin	F^1^	5'-ACGTCTCACTGGATTTCGAGCAGG-3'	NM_205518	298
	R^2^	5'-TGCATCCTGTCAGCAATGCCAG-3'		
GAPDH	F	5'-GAGGGTAGTGAAGGCTGCTG-3'	NM_204305	113
	R	5'-CATCAAAGGTGGAGGAATGG-3'		
NF-kB 1	F	5'-GAAGGAATCGTACCGGGAACA-3'	NM_205134	131
	R	5'-CTCAGAGGGCCTTGTGACAGTAA-3'		
IL-1β	F	5'-GCTCTACATGTCGTGTGTGATGAG-3'	NM_204524^3^	80
	R	5'-TGTCGATGTCCCGCATGA-3'		
IL-6	F	5'-AGGACGAGATGTGCAAGAAGTTC-3'	NM_204628	78
	R	5'-TTGGGCAGGTTGAGGTTGTT-3'		
IL-18	F	5'-CACTGTTACAAAACCACCGC-3'	NM_204608d^4^	213
	R	5'-CTTAAAAGCCTTGGAGCTGC-3'		
TL1A	F	5'-CCTGAGTTATTCCAGCAACGCA-3'	NM_001024578^5^	292
	R	5'-ATCCACCAGCTTGATGTCACTAAC-3'		
TLR-4	F	5'-TGCACAGGACAGAACATCTCTGGA-3'	NM_001030693^6^	347
	R	5'-AGCTCCTGCAGGGTATTCAAGTGT-3'		
TLR-15	F	5'-TGCTGCCACATTTGGAAGATC-3'	NM_001037835	131
	R	5'-GATCGGTGCTCCACACAAGTC-3'		
TRAF6	F	5'-AGTAAATACGAGTGCCCGATCT-3'	CK607050	176
	R	5'-TTAGCGAAGTTGTCTGGAAAAA-3'		
MyD88	F	5'-AAGTTGGGCCACGACTACCT-3'	NM_001030962	216
	R	5'-CTGCTGCTTCCTTCGTAAGT-3'		

^1^Forward primer. ^2^Reverse primer. ^3^From Ferro *et al.*, 2004. ^4^From Sadeyen *et al.*, 2004. ^5^From Takimoto *et al.*, 2005. ^6^From He *et al.*, 2006.
